# Education and Training in Psychiatry: Challenges and Consequences of the Last Two Years, Future Perspectives and Actions Needed

**DOI:** 10.1192/j.eurpsy.2022.51

**Published:** 2022-09-01

**Authors:** K. Başar

**Affiliations:** Psychiatric Association of Turkey, Department Of Psychiatry, Hacettepe University, Ankara, Turkey

## Abstract

Pandemic-related extraordinary work burden on all healthcare professionals had a greater toll on younger colleagues, especially residents in diverse medical fields. Although psychiatry residents’ degree of engagement in COVID-19 related services depends on the state’s and institution’s management of the crisis, it has often led to a significant interruption in the schedule of the training requirements. This interruption also was a consequence of the change in provision of the psychiatric services during the pandemic, such as a decrease in the size of the inpatient clinics, lower number o patients in the outpatient services, fewer ECT. These changes made it more difficult to fulfill the training requirement, which was adopted in some countries. Even when the tasks related to the training were accomplished the quality was difficult to determine. Furthermore, most of the educational activities were switched to online format due to pandemic-related precautions, a change that persists in some activities. Psychiatry education and training, not only postgraduate but also during medical school, requires observation and direct engagement. The switch from face-to-face to online in psychiatry training, including supervision, and service provision constituted a major challenge in psychiatry education. This required further adaptations by the teaching staff as well as the trainees, in their methods of teaching, learning, and interaction. The assessments of competencies presented another challenge for the psychiatry training. Despite all, the introduction of online education provided increased diversity in resources and in some situations increased access. Some challenges required modifications, whereas some beneficial changes are there to maintain.

**Disclosure:**

No significant relationships.

